# Functional blindsight and its diagnosis

**DOI:** 10.3389/fneur.2024.1207115

**Published:** 2024-02-07

**Authors:** Timothy Joseph Lane, Tsan-Hon Liou, Yi-Chia Kung, Philip Tseng, Changwei W. Wu

**Affiliations:** ^1^Graduate Institute of Mind, Brain, and Consciousness, Taipei Medical University, Taipei City, Taiwan; ^2^Brain and Consciousness Research Centre, Taipei Medical University, Taipei City, Taiwan; ^3^Institute of European and American Studies, Academia Sinica, Taipei City, Taiwan; ^4^Department of Physical Medicine and Rehabilitation, School of Medicine, College of Medicine, Taipei Medical University, Taipei City, Taiwan; ^5^Department of Physical Medicine and Rehabilitation, TMU Shuang Ho Hospital, New Taipei City, Taiwan; ^6^Department of Radiology, National Defense Medical Center, Tri-Service General Hospital, Taipei City, Taiwan; ^7^Taiwan Institute of Neuroscience, National Yang Ming Chiao Tung University, Hsinchu, Taiwan; ^8^Department of Psychology, National Taiwan University, Taipei City, Taiwan; ^9^Research Center for Mind, Brain and Learning, National Chengchi University, Taipei City, Taiwan

**Keywords:** blindsight, consciousness, Riddoch Syndrome, P300, fMRI, hypercapnia challenge, self-relatedness, akinetopsia

## Abstract

Even when brain scans fail to detect a striate lesion, functional evidence for blindsight can be adduced. In the aftermath of an automobile accident, JK became blind. Results of ophthalmic exams indicated that the blindness must be cortical. Nevertheless, multiple MRI scans failed to detect structural damage to the striate cortex. Prior to the accident JK had been an athlete; after the accident he retained some athletic abilities, arousing suspicions that he might be engaged in fraud. His residual athletic abilities—e.g., hitting a handball or baseball, or catching a Frisbee—coupled with his experienced blindness, suggested blindsight. But due to the apparent absence of striate lesions, we designed a series of tasks for temporal and spatial dimensions in an attempt to detect *functional* evidence of his disability. Indeed, test results revealed compelling neural evidence that comport with his subjective reports. This spatiotemporal task-related method that includes contrasts with healthy controls, and detailed understanding of the patient's conscious experience, can be generalized for clinical, scientific and forensic investigations of blindsight.

## Highlights

- Blindsight can occur even in the absence of detectable striate cortex lesions.
- Spatiotemporal tasks can be employed to adduce functional neuroimaging evidence of blindsight.
- Unlike other blindsight patients, JK's residual conscious experiences impede rather than facilitate motor performance.

## Introduction

For those rare cases of human bilateral blindsight, anatomical brain images invariably detect post-chiasmatic lesions (1, 2). Blindsight is a type of cortical blindness that is distinctive because patients retain the ability to perform appropriate saccades, visually discriminate among stimuli, or behaviorally react to objects in motion, despite having suffered damage to the striate cortex, damage that can impede the ability to form conscious visual percepts (3–5). Although some authors have suggested that the striate cortex might not be necessary for conscious vision (6–11), for purposes of this article, we do not engage that dispute. According to the widely held view of blindsight, visual stimuli can be processed, despite damage to the striate cortex, because there are alternative visual pathways that do not involve consciousness (12). But it remains unclear what types of neuroimaging data can be adduced to provide evidence for cases of blindsight wherein the striate cortex appears to be intact.

Our investigations focused on a patient (JK) who presented as cortically blind. In the aftermath of a November 2009 automobile accident, JK soon discovered that his vision had been affected. One month after the accident, clinical examination revealed that he could not even distinguish light from dark, bilaterally. Nevertheless, JK, an outstanding athlete, especially adept at handball, retained an ability to respond behaviorally to stimuli (e.g., successfully hitting a ball) in a manner that suggested residual vision, despite the lack of conscious visual percepts. Strikingly, unlike all prior investigations of blindsight (13), multiple 3T MRI scans over a decade evinced no anatomical evidence of a striate lesion.

From 2010 to 2019, using Visual Evoked Potential (VEP), neurologists in Taiwan consistently found JK's vision to be severely impaired. Accordingly, in 2019 a neurologist working with our team at the Brain and Consciousness Research Center also used VEP to yet again confirm this impairment (see Supplementary Figure S1). The neurologist reported that JK's eyes could move freely, and that the light reflex for both eyes was normal; these findings imply that the pathway from retina to chiasma, optic tracts, midbrain, and back to pupils via the oculomotor nerve is unimpaired. VEP results, however, yet again evinced severe impairment (Y-H Huang, personal communication, July 10, 2022).

A caveat is necessary here: it should be kept in mind that eye movement and light reflex alone are not sufficient to guarantee that the visual pathway is intact. It remains empirically possible that symptoms are still caused by problems of input to the cortex, e.g., the result of optic radiation lesions (14). Nevertheless, VEP findings, eye movement and light reflex—when coupled with our *functional* neuroimaging discovery of striate cortex impairment—suggest that neither optic radiation nor other lesions to visual pathways are the main cause of JK's symptoms.

The principal problem addressed here can be stated thus: because JK's striate cortex seems intact, what brain imaging methods can be used to probe and adduce neural evidence of his blindsight? The importance of getting clear about this problem is accentuated by the fact that JK was accused of fraud in both civil and criminal courts. His athletic ability, forensic unfamiliarity with the concept “blindsight”, and the atypical nature of his condition—viz. failure to find evidence of striate structural damage—combined to contribute to his legal problems. Indeed, JK's case was first brought to our attention in the hope that we could devise a scientifically sound method for assessing JK's neural damage, both for legal and clinical purposes. Neither VEP nor structural imaging can provide adequate evidence that accounts for JK's neural damage cum behavioral capabilities. Therefore, our challenge has been to develop neuroimaging methods suited to the task of adducing evidence when structural imaging and clinical methods prove to be inadequate.

With this objective in mind, we designed a protocol that includes three components: the first concerns adequate characterization JK's blindsight. Several conceptual-empirical concerns motivate this component: the challenge of distinguishing blindsight from severely impaired conscious vision (15, 16), methodological problems pertaining to subjective report (17) and response bias (18–20), theoretical disputes pertaining to the nature of consciousness (21, 22), recognition that blindsight exhibits much variety (5, 23), and overlap between blindsight and Riddoch's Syndrome (10, 11, 24, 25). To provide a fine-grained description of JK's blindsight, therefore, we reviewed the entirety of his clinical history, conducted a detailed, semi-structured interview, and included assessment with the Sensation Awareness Scale (26, 27). More recently we conducted a follow-up interview in order to better understand the 14-year arc of change in his perceptual-motor capabilities.

Next, because previous MRI scans evinced no indication of striate lesions and because VEP only helps determine whether vision is impaired, we compared JK to healthy controls (HC), using tasks and multimodal methodologies. The aim is to search for *functional* neural evidence of his blindsight. Accordingly, our second component investigated the temporal dimension, employing an event-related potential (ERP) probe.

Then for the spatial dimension, we employed MRI. First we sought to confirm previous structural MRI clinical reports that failed to find evidence of a striate lesion. Next, we employed functional magnetic resonance imaging (fMRI) coupled with task so that, just as with ERP, we could probe for functional evidence of neural damage. The combined purpose of ERP and fMRI then is to determine whether temporal and spatial evidence converge in helping to provide functional evidence of JK's blindsight.

Finally, we acknowledge that there are numerous unresolved conceptual issues in this vicinity. Among other things it has been argued that Riddoch Syndrome and Type 2 Blindsight are indistinguishable (28) or that some cases thought to be Blindsight are more accurately described as cases of degraded sight (29). In the latter case, the suggestion is that behavioral performance correlates with some degree of awareness. Importantly, a possibly unique characteristic of JK's blindsight is that although he retains a (severely) limited capacity for conscious vision, for him attempts to utilize such limited *conscious awareness impedes athletic performance*.

Indeed, part of the problem with characterizing the phenomenon we are trying to explain is that different reports concern different patients or the same patient at different points in time; each patient has distinctive characteristics that evolve across time. But of equal or greater importance, yet more work needs to be done explicating each of the relevant concepts (23). For purposes of this manuscript we have not attempted to adjudicate among the many nuanced conceptual issues in this vicinity. Instead, we focus on the specific clinical problem confronted by JK—no obvious striate structural damage coupled with apparent absence of the conscious visual experiences that typically accompany athletic performances of which he is capable. JK's distinctive experience of blindsight—including the fact of residual awareness disrupting athletic performance—is at once a clinical, scientific and forensic conundrum.

Although our purpose is to determine whether functional evidence can be adduced, we take it to be a first principle in biology that structure determines function. That is, our ambition is not to challenge this principle. Instead, we believe it far more likely that the absence of observable damage reflects methodological and resolving limitations. In this regard, the proper way to understand our investigation is, given these limitations, how can a proper scientific and clinical assessment of blindsight proceed? Here we apply it to JK, while acknowledging that a deeper explanation likely involves the structural level (30). In principle, however, our methodology could be applied generally to investigations of blindsight in all its varied manifestations.

## Materials and methods

### IRB and participants

This study was approved by the TMU-Joint Institutional Review Board (N202111025). Written consent was obtained from JK and from the two HCs. Although JK cannot read, the IRB was explained to him in the presence of a witness. He signed, as did the witness. Moreover, JK was provided with a written version that is translatable into an auditory version.

### Patient history and subjective report

On November 24, 2009, when he was 20 years old, JK was involved in an automobile accident. He was rendered unconscious and upon recovery of consciousness realized that he had suffered numerous internal and external injuries. At first the medical team charged with his treatment attended mostly to external injuries, especially from the waist down, as well as limb paralysis and numbness. On the day after the accident, JK was globally conscious, but due to a concussion also suffered during the accident, he felt dizzy and nauseous. At that time, however, he was not aware of any impairment to the contents of visual consciousness.

On the second or third day of his hospitalization he gradually came to realize that his vision had been affected. He describes his vision at the time as being somewhat fuzzy, or “watery,” similar to what one normally experiences upon awakening, when teary, or when tired. At least though at the time by “blinking” and with effort he could still bring objects into focus. By the fourth day, however, he recalls from discussions with his mother about noise outside of his hospital room or about the timing of meals, that he had trouble distinguishing day from night. He realized that even when his eyes were open he experienced only darkness.

Over the course of a few days, however, he recovered the ability to detect light as well as an indistinct sense of bright colors. He recalls that by staring at an object for several minutes he could sometimes detect colors, albeit not distinct shapes. And when in a car he noticed a marked difference between the visual experience of looking out and looking in, the bright outside light enabling some visual experience. Indeed, one set of tests conducted prior to our investigation of JK suggested that he retained a slight degree of residual function for his lower left visual field. These tests and experiences, along with an ophthalmologist report that his eyes were undamaged and that problems with vision might be due to prolonged immobility, combined to give him confidence that his vision was recovering, in concert with his mobility.

Although a January 2010 Technetium-99m PET examination did suggest occipital abnormality and though he continued to experience headaches, along with rehabilitation to recover mobility he persisted in trying to recover vision. Consistent with the test that revealed slight lower left field residual function, he tried to stimulate vision by holding his left eye close to the page of a book. By persisting, under bright light, he could sometimes consciously distinguish between blank spaces and written words, at least in that the latter appeared shadowy. Moreover, he would bounce a yellow handball off the wall and try to catch it as it rebounded. Nevertheless, though his mobility has recovered to the extent that he now exhibits athletic grace when he moves, his vision has not exhibited conspicuous improvement.

As for his residual conscious vision in 2023, it has distinctive characteristics. First, he continues to be able to distinguish light from dark, but this capacity is not self-evidently associated with the formation of visual percepts. That is, when asked to introspect and assess his conscious contents, JK emphasizes that his mood is affected by the difference between light and dark. Darkness tends to be associated with anxiety, whereas bright light has a calming effect. JK reports that the experience of light is comforting; darkness induces anxiety.

A second characteristic—possibly unique among reported cases of blindsight—related to distinguishing light from dark, is that *his conscious percepts seem to operate on an intrinsic delay setting*. As an example, JK notes that when he is in a car and entering a tunnel, he can tell by sound that the tunnel has been entered. Nevertheless, light lingers. On the other hand, likewise by sound he knows when he's emerged from the tunnel. But darkness lingers. Similarly, when trying to use vision to help respond to the bright yellow handball that he uses for practice, he might detect a yellow patch, but even after the ball has hit him in the forehead, the yellow patch continues to hover, as though it remains a foot or so away. In other words, whereas for other patients who suffer from blindsight residual awareness facilitates motor activity, for JK residual awareness is an impediment.

For a time JK tried to incorporate visual experience into his ability to hit a bright yellow handball. But these efforts were counter-productive. Because of the temporal delay—the patch of yellow continuing to hover after the ball had already struck him—in order to successfully strike the ball, conscious visual percepts had to be ignored. In other words, even when the hovering color patch appears, in order to perform effectively, JK must rely on reflexive, unintentional motor responses. JK also indicates that he seems better able to respond to some quickly moving objects, but these instances are not necessarily accompanied by color patches. In short, his responses to external stimuli in motion tend to be accurate when they are reactive, not intentional, *and not when guided by conscious visual experience*.

A third characteristic is that by intensely staring at an object under bright light, he seems to be able, over time, to form partial visual percepts. As an example, he has several times demonstrated this by holding his left eye within an inch of an open book. “Visual” experiences are not immediate, nor can they always be successfully achieved. But by subtle angle adjustments and intense gazing, sometimes he achieves limited vision, at least in one sense: when successful he can distinguish between bright and shadowy areas. He is thereby able to infer where on the page are written words.

During an interview with JK conducted on October 3, 2023 we probed the 14-year arc of change in his perceptual-motor capabilities, focusing on his ability to catch Frisbees and hit baseballs. He commented that even several years ago, at a time when he exerted more effort hoping to recover conscious vision, the ability to catch a Frisbee successfully required 3 months of practice and even then his success rate was only about 10%. As for hitting a baseball, 3 months of practice were also required but his success rate was substantially higher. The difference in performance success he believes is due to greater standardization of pitching motions for baseball, thereby enabling him to better anticipate release time of the projectile. He emphasized that in order to catch or hit successfully, it is necessary that he be properly oriented to the location and timing of the object thrown in his direction. Typically, the person holding the Frisbee or baseball would call out to JK indicating that the object was about to be thrown. JK also noted that he was only able to be successful when he remained mostly stationary; when attempting to move more than a step or two, he could not be successful.

Here too, however, conscious visual percepts are only achieved under extremely bright light and the experiences are limited to sensations of color, projectiles lacking distinctive shapes. Previously he was more driven to use practice as a means of stimulating regeneration of the capacity for conscious vision. Fourteen years of failure to improve beyond faint sensations of color under bright light, however, have lessened his motivation to devote time to practice. Accordingly, his ability to catch or hit objects regresses.

As is the case with all blindsight patients (5), JK's phenomenology is nuanced. But he does not suffer from Type 1 blindsight; he is not wholly lacking any visually-relevant conscious experiences. Instead, his blindsight seems more aptly characterized as Type 2. That is, something is felt but those feelings are not self-evidently visual (31–33). Moreover, in certain respects, notably his ability to consciously see the color of moving objects, some of his symptoms are suggestive of Riddoch Syndrome (11).

When making a general assessment of his capacity for visual consciousness, we queried him using the Sensation Awareness Scale, SAS (26). SAS comprises these items: (3) “I do not see anything,” (4) “I don't think that I see anything, but I am not certain,” (5) “I feel something,” (6) “I see something,” or (7) “I clearly see something and can identify it”. He affirmed that (3), (6), and (7) do not describe his experiences. Instead, based upon his introspective assessment, he believes that sometimes (4) and sometimes (5) best describe his visual experiences. It should be noted that the SAS has sparked some controversy pertaining to conceptual and methodological issues (27, 34), so while not wishing to weigh JK's responses to SAS too heavily, to a first approximation his blindsight seems more aptly described as Type 2 rather than Type 1, while also being suggestive of Riddoch Syndrome.

In addition to JK's medical problems, because recovery from external and internal injuries was accompanied by recovery of a measure of athletic prowess, he was accused of fraud. The basis of the accusation being that if he were really blind, he would not be able to perform athletics. Failure to detect a striate lesion compounded his problems. It was in this context that we were contacted by Taiwan's Control Yuan, a branch of Taiwan's central government that serves as an ombudsman, with powers that include oversight of the judiciary. Because JK had been found guilty, in a contentious ruling, we were asked to investigate, to see whether or not we could develop a scientifically sound method for determining whether he is indeed suffering from blindsight. Specifically, we designed two experiments, ERP and fMRI, for distinct purposes: ERP was adopted to probe JK's conscious vision through a self-awareness paradigm, and fMRI was used to measure JK's brain-function integrity, as it relates to vision but without explicitly mentioning vision.

### Event-related potential, P300

#### Rationale

P300 is regarded as a component of memory recognition, in experimental (35, 36), medical (37), and even forensic contexts (38–40). Crucially, specifically relevant to our investigation, according to the Global Neuronal Workspace Theory (GNWT), it can also be a signature of conscious perceptual processing (41, 42). Typically it is induced in the context of an “oddball paradigm,” wherein a familiar object/person is intermixed with unfamiliar stimuli (ratio usually from 1:4 to 1:6) and presented to participants in sequential, randomized order. The stimuli can be delivered visually or verbally. After seeing or hearing the familiar or self-related stimulus (43), participants usually evince stronger ERP amplitude during the 300–600 ms post-stimulus window—namely, the P300 response.

Clinically, P300 and self-related stimuli have been used to investigate levels of or global consciousness, helping improve prognostic and diagnostic accuracy (44–47). Improved diagnosis or prognosis is accomplished by assessing reactions when familiar or self-related stimuli are intermixed with unfamiliar ones (43, 48, 49). And, in forensic contexts, the oddball paradigm can be modified such that crime-related objects only known to the culprit (e.g., a murder weapon) are intermixed with unfamiliar objects. In this way, by hypothesis, only the culprit should evince stronger P300 when a crime-related object appears (50). In short, this approach is well-suited to investigation of both veracity and conscious perception.

#### Task and procedures

**Figure 1:**
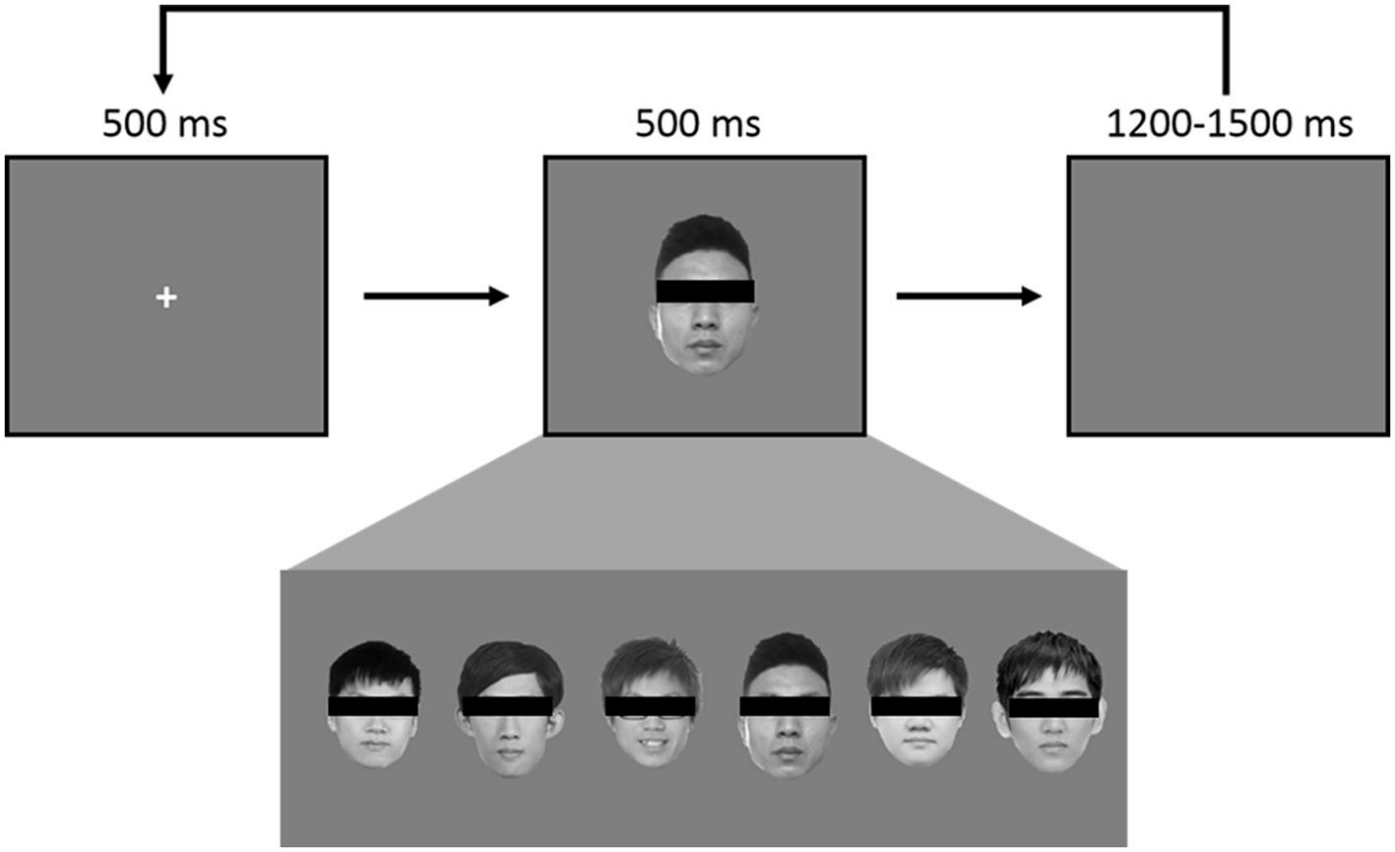
Each face was used 60 times, in randomized order, for a total of 360, such that there was a 1/6 probability of JK's self-own-face appearing in any given instance. During the experiment each face was visible.

The experiment consisted of 360 trials using a pool of 6 grayscale faces to create the classic “oddball” paradigm. In every trial, a face is shown in the middle of the screen, roughly the size of 11° by 12° in visual angle. A pool of 6 faces was used: one belonged to JK (Figure 1, third face from the right) and one to the HC (Figure 1, first face from the left). Each face appeared 60 times throughout the entire experiment, and the order was randomized (Figure 1). At the start of every trial, a 500 ms fixation cross was displayed at the center, followed by a face image for 500 ms, and concluding with an inter-trial interval (ITI) jittering randomly between 1,200 ms and 1,500 ms. A break was provided every 120 trials.

#### Electroencephalography recording

EEG was continuously recorded from 27 Ag/AgCl electrodes according to the 10/20 system, with a reference electrode between Fz and Cz. All electrodes were mounted on the BrainCap electrode cap (Brain Products GmbH, Munich, Germany). All signals were amplified using BrainAmp amplifier (Brain Products, Munich, Germany). The signals were digitized at a sampling rate of 1,000 Hz. Two sets of electrodes were placed on the upper and lower sides of the right eye, and the canthi of both eyes to measure participants' vertical and horizontal eye movements.

#### Event-related potentials

A 0.5 Hz high-pass filter was first applied to the EEG data to remove the trend of the continuous EEG data. The ocular artifacts from continuous EEG data were removed by using independent component analysis [ICA, (51)]. The oculo-corrected continuous EEG data were then segmented into epochs: from 100 ms before the stimulus onset to 800 ms after onset, then offline re-referenced to the average of electrodes at the left and right mastoids (M1 and M2). A digital low-pass filter of 30 Hz (24 dB/octave) was applied to filter out high frequency noise. Baseline correction was executed using a pre-stimulus interval. Epochs with artifacts fluctuating over ±100 μV. Remaining trials were averaged according to stimulus type (i.e., self vs. other).

## Results

In the EEG task, each subject saw their own self-own-face 60 times (viz., “self” condition) and five others' faces 300 times (“other” condition). The HC had 56 artifact-free trials in the self-condition and 293 artifact-free trials in the other condition. JK had 60 artifact-free trials in the self-condition, and 290 artifact-free trials in the other condition. We averaged artifact-free trials according to stimulus type (self vs. other).

**Figure 2:**
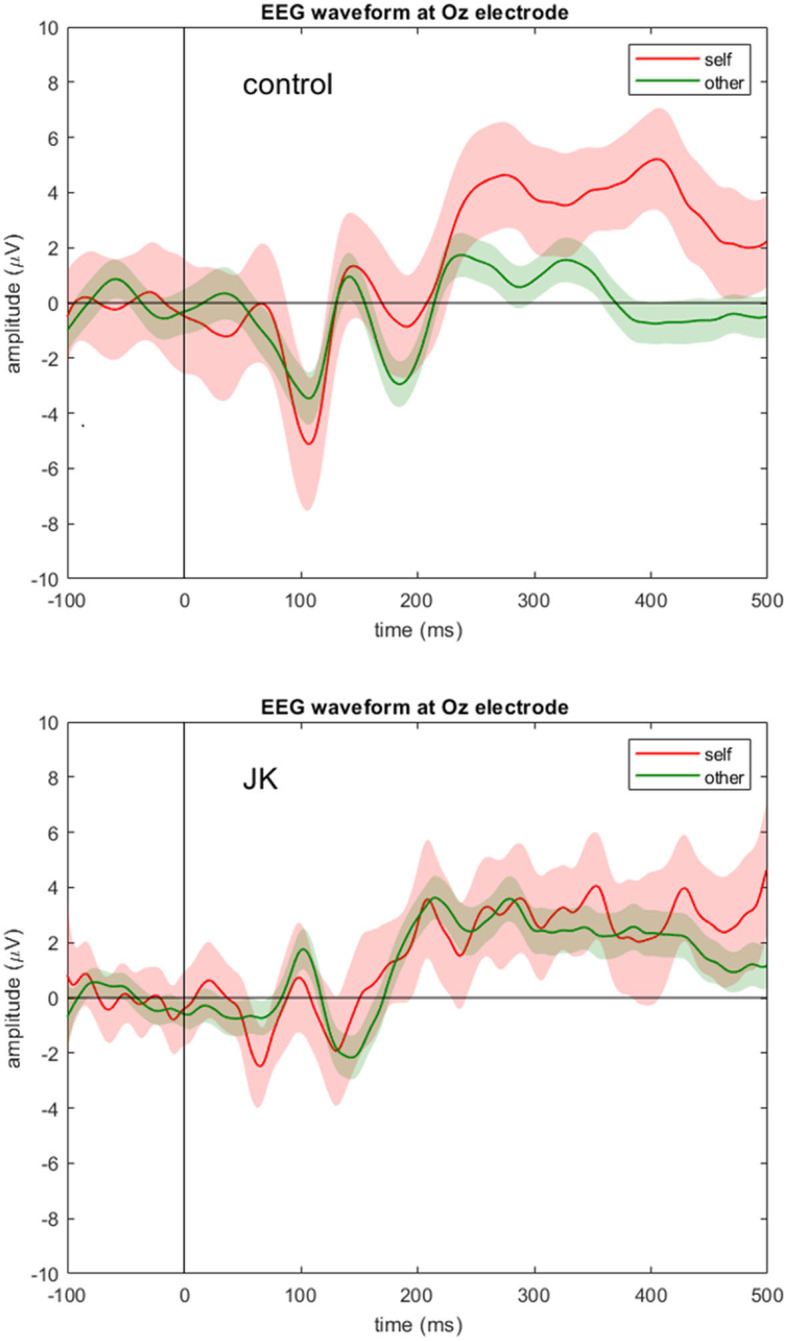
EEG waveform at Oz electrode. Grand averages (solid lines) and 95% confidence intervals (shaded areas) at Oz electrode for the self (red) and other (green) condition for the control subject (**top**) and JK (**bottom**). Compared to control, JK shows abnormal, but not flattened, visual responses from Oz.

Figure 2 shows the grand average waveforms of self vs. other condition in both the control subject and JK at Oz. For the HC, we observed a negative peak amplitude at 107 ms after stimulus onset (peak amplitude: −5.12 μV in self condition, and −3.47 μV in other condition). The N1 component is therefore apparent in both conditions in the HC. In contrast, JK had a weaker negative trend after stimulus onset (the first negative peak amplitude: −2.49 μV in self condition, and no clear peak in the other condition), that looks like a compressed (and slightly noisier) version of HC. This was not only true for Oz (Figure 2), but O1 and O2 as well (not shown). This observation is consistent with JK's clinical VEP results, where abnormal early visual waveforms are observed from the visual cortex yet these waveforms are not identifiable as standard P1 or N1. Such compression in VEP may signify poor bottom-up signals, or lack of feedback from higher visual areas, or both; we cannot be certain at this time. But this is indeed why we argue that later components like P300 seem better suited to investigations of blindsight, since we know that conscious visual signals are more sluggish than are non-conscious visual signals (52).

**Figure 3:**
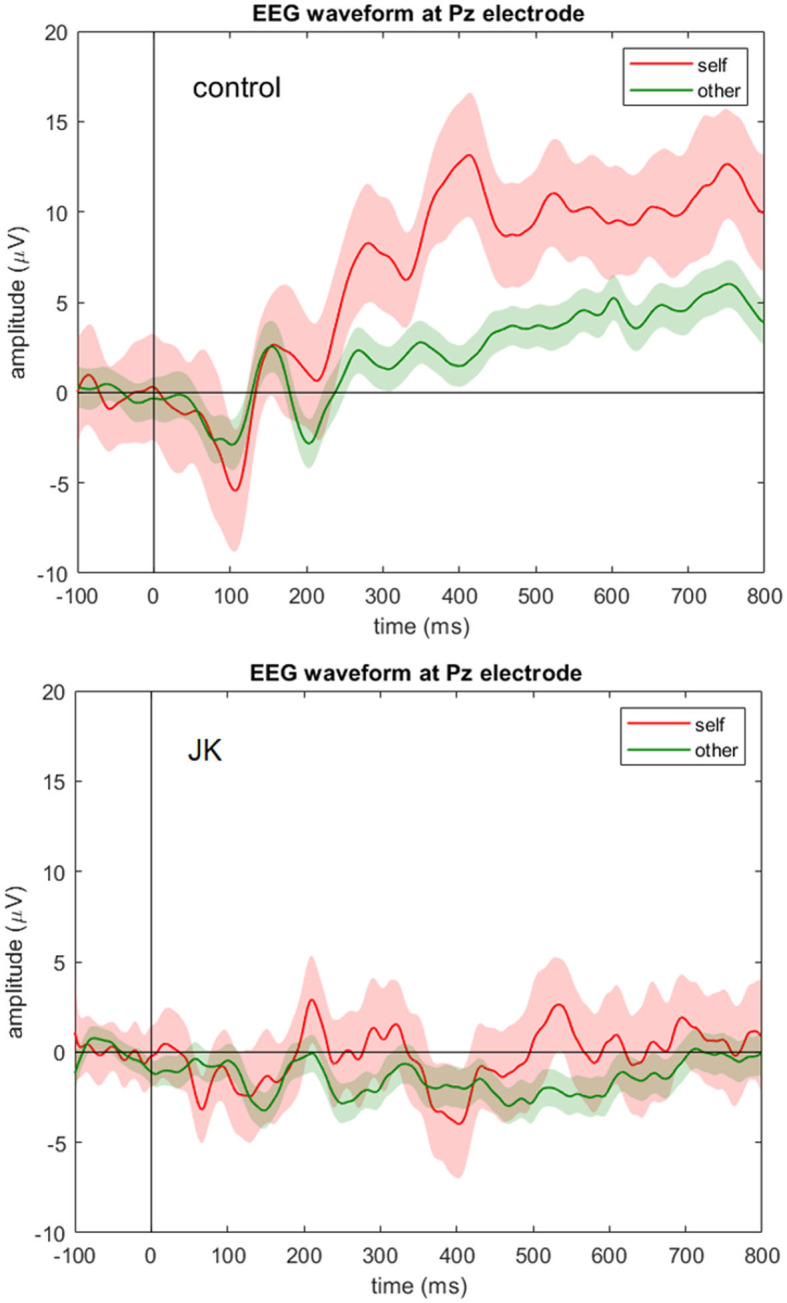
EEG waveform at Pz electrode. Grand averages (solid lines) and 95% confidence intervals (shaded areas) at Pz electrode for the self (red) and other (green) condition for the control subject (**top**) and JK (**bottom**). Compared to control, JK shows a flattened ERP waveform at Pz with no visible P300 component for either self-own-face or others' faces.

To investigate whether JK was consciously aware of what he saw, we compared the P300 component between him and the HC, and also between self and other conditions. Figure 3 shows the grand average waveforms of self vs. other condition in control subject and JK, at Pz. In the HC, we found that the 95% confidence intervals from the self and other conditions began to diverge from 247 ms after the face image onset, which just is the P300 component. For JK, grand average waveforms with 95% confidence intervals from self and other conditions overlapped throughout most of this time window, except for a very narrow window from 521 to 550 ms (29 ms). A direct test between self and others in all electrodes also evinces sporadic openings with significant differences, but all such openings were <51 ms, some even as short as 1 ms. Therefore, we conclude that there was no visible P300 from JK's data, neither for his self-own-face nor for others' faces, and that there was no solid evidence of a delayed P300 or any other forms of self vs. other differences either.

### MRI/fMRI

#### Rationale

The magnetic resonance imaging (MRI) procedures were designed to investigate the functional impairment of JK's conscious vision. Procedures included structural images, a vision-related task (flashing checkerboard stimuli), and tasks unrelated to vision (breath holding and resting state). Among these MRI imaging techniques, the structural images allowed for examination of cortical structure integrity, with special focus given to the striate cortex; the vision-related task was used to determine whether visual stimuli were being processed; and, the tasks unrelated to vision were used to assess the *functional* integrity of JK's visual cortex. One reason for including tasks unrelated to vision was to control for the possibility that JK might have intentionally concealed his capacity for conscious vision.

#### Structural integrity

One of the prosecution's arguments against JK is that his brain did not appear to be structurally impaired, especially in his visual system. And since ophthalmology exam results were all negative, his conscious visual perception should be intact. It is plausible, however, that even seemingly intact brain structures may exhibit functional abnormalities. Structure determines function, but there are limits to current methodology and resolving power of brain imaging equipment.

To adduce evidence for this conjecture—function and task will reveal what structure does not—we began by reassessing JK's previous MRI scans, scans that were conducted at several hospitals. We retrieved four of these MRI anatomical datasets from examinations carried out over a decade−2010 Jan, 2011 Nov, 2016 May, 2019 Jan—the last of these having been carried out by our Brain and Consciousness Research Center. Because imaging parameters and contrasts for the 2011 and 2016 data sets were incompatible, we omitted those. Accordingly, we only present cortical thickness results from the January 2010 and January 2019 scans. For these two we estimated the cortical thickness of gray matter based upon T_1_-weighted anatomical images (53).

#### Functional integrity

Based on the blood-oxygenation-level-dependent (BOLD) principle, fMRI has been widely applied to noninvasive, indirect inference of the underlying neural activity (54). A visual task that employs an 8-Hz flashing checkerboard is a standard, robust method for inducing striate cortex activity; this task can help to indicate whether visual stimuli are being processed at the first cortical stop (55). Typically, after activity in the striate cortex is triggered, visual information passes along the dorsal and ventral pathways to the frontal eye field, enabling emergence of visual consciousness.

In other words, intact conscious visual perception in healthy subjects results from recursive brain activity that engages posterior and anterior regions (56). If any impairment occurs along this network, visual consciousness may be impaired or blocked. Therefore, in order to determine whether and how network activity might be arrested in JK, we adopted two approaches: a breath-holding and a resting-state fMRI, to measure integrity of cerebrovascular reactivity (CVR) as well functional connectivity of the visual network (57, 58).

The breath-holding task induces a transient hypercapnia status, followed by fMRI detectable global hyperemia throughout the cortex. The CVR induced by breath-holding makes it possible to assess autoregulation in cerebral vasculature. Use of this method enables us to measure the transient hyperemia response in JK's visual cortex. As for the resting-state fMRI, it enables us to assess the temporal correlation across distant brain regions. High temporal correlation of time curves is taken to imply “functional connectivity” (FC), and variation in resting-state functional connectivity (RSFC) has been shown to be associated with certain neurological diseases, psychiatric disorders, and states of consciousness (58, 59). For JK we employed the resting-state fMRI to assess the visual network's RSFC.

#### Procedure of MRI experiment

Our Centre's MRI experiment was conducted on a 3T MR750w scanner (GE, Milwaukee, WA, USA) on January 27, 2019. Two participants underwent the imaging protocol: JK (male, 29 years old), and one HC with normal vision (male, 39 years old). To record physiological signals, the participants wore the physiological monitoring unit (PMU, including pulsation and respiration); to convey messages to the experimenter, they wore a headset with microphone. Visual stimuli were projected with E-Prime (Psychology Software Tools, Pittsburgh, PA, USA). MRI-compatible goggles and a mounted eye-tracker were used during the visual task (VisuaStim Digital, Resonance Technology Inc., Northridge, CA); the eye-tracker was used for real-time monitoring of the participants' pupil orientation and in order to ensure participant compliance with the instruction to keep eyes open. To minimize artifact caused by speech, thermoset plastics were used to restrain participants' head motion.

#### MR imaging protocols

The high-resolution T_1_-weighted anatomical image was obtained through use of an Axial FSPGR BRAVO sequence with 256 × 256 × 124 matrix size; 0.94 × 0.94 × 1.2 mm^3^ in-plane resolution; 450 ms inversion time; repetition time (TR) = 8.49 ms, echo time (TE)= 3.25 ms; flip angle (FA) = 12°; pixel bandwidth= 244.1 Hz/pixel; NEX=1. Total scan time was 5 min.

The functional sessions shared the same geometric settings: thirty-seven axial slices (FOV = 220 × 220 mm^2^, 64 × 64 in-plane matrix size, and 3.4 mm thickness without gap), acquired in an interleaved manner, and aligned along the anterior commissure–posterior commissure (AC-PC) line, with whole-brain coverage. The scan protocol employed a single-shot, gradient-echo-based echo-planar imaging (GE-EPI) sequence with imaging parameters set thus: TR = 2 s, TE = 35 ms, FA = 84°. Using these settings, the two participants performed two tasks: flashing checkerboard and a breath-holding task. The first tested brain activity in response to external visual stimuli; the second, probed functional integrity of the visual network. Likewise using these settings, spontaneous resting state activity was measured, also in order to help assess functional integrity of the visual network.

#### Visual task

For the visual task, participants were asked to open their eyes and passively view a full field screen. While scanning, the screen exhibited two conditions: a “stimulation” and a “fixation” period. During stimulation, a black and white circular checkerboard pattern flashed at 8-Hz with the spatial frequency of 1.67 cycles per degree; during fixation, a static cross pattern was presented at the center of the screen, in an interleaved manner (60). The block design paradigm started with a 20-s resting period, followed by six cycles of 20-s stimulation and 20-s resting periods. The acquisition time lasted 260 s, comprising 130 scans.

#### Resting state

One six-minute eyes-closed (EC) resting-state session was measured. Participants were instructed to close eyes, remain still head position and avoid thinking of anything in particular (viz., let their minds wander). To avoid falling asleep, participants were asked to press the pneumatic ball at the cessation of the scanning acoustic noise, indicating they maintained wakefulness for this session. Acquisition time lasted for 420 s and comprised 210 scans.

#### Breath-holding

For the breath-holding experiment, a block-design was used. It comprised 5 blocks, for a total scanning time of 5 min. Participants were instructed to breathe naturally for the first 28 s. Next, auditory instructions were given through the microphone: “Exhale” presented on the screen for 2 s; the participants were instructed to exhale completely. At 30 s the “hold your breath” instruction was given, and they started to hold their breath for 15 s (61). This was followed by the instruction “breathe naturally,” which was measured for 43 s. Another block followed, and the sequence continued until five blocks were completed. During the scan, respiration signals were monitored to ensure participant compliance. Total acquisition time for 166 scans lasted 324 s.

#### Other MR images from different sites

To trace the longitudinal changes of the cortical thickness of JK, we retrieved additional anatomical images from scans carried out at another hospital in January of 2010. The T_1_-weighted anatomical image was obtained from a Siemens Verio 3T scanner using an Axial 2D-FLAIR sequence with 276 × 320 × 22 matrix size; 0.72 × 0.72 × 6.5 mm^3^ in-plane resolution; 860 ms inversion time; TR = 2,000 ms, TE = 9 ms; FA = 150°/90°; pixel bandwidth = 260 Hz/pixel; NEX=1. Total scan time was 173 s.

#### Data analysis

Brain cortical thickness was calculated with FreeSurfer v5.3.0 [http://surfer.nmr.mgh.harvard.edu/, (53)]. Input images were processed with the correction of magnetic inhomogeneity, skull strip, and intensity normalization, then segmented to gray and white matter. The brain surfaces were then tessellated into many triangles, with each cluster of six adjacent triangles taken as a vertex. The pial surface and the gray-white matter boundary were reconstructed with image intensity and location information. The distance between gray-white was then calculated from point to point and defined as cortical thickness. We then extracted the thickness of the visual cortex based on the Destrieux Atlas with the gyrus and sulcus parcellation [an automated labeling system for subdividing the human cerebral cortex on MRI scans into gyral-based regions of interested (62)].

The functional datasets were preprocessed with slice-timing correction, motion correction, rigid-body normalization into the Montreal Neurological Institute (MNI) template space, and spatial smoothing (smoother kernel= 6 mm) using IClinfMRI (63) as well as Analysis of Functional Neuroimaging (AFNI) software packages (64). After the standard preprocessing, the visual-task and CVR data underwent general linear model (GLM) analysis, using each responded trials convolved with canonical hemodynamic response function without filter, to retrieve the brain activation. In addition, the resting-state fMRI data underwent linear detrend and temporal filtering (0.01–0.08 Hz). Finally, we prescribed the seed points of the target brain regions (primary visual cortex in calcarine fissure and fusiform gyrus) in the MNI coordinate and conducted the seed-correlation analysis to access the RSFC maps. Quantitative comparison was carried out for regions-of-interest (ROIs) analysis, ROIs including: including: bilateral calcarine fissure, superior/middle/inferior occipital gyri, fusiform, precentral and postcentral cortices. These were selected from the Automated Anatomical Labeling (AAL) template (65).

#### MRI results

**Figure 4:**
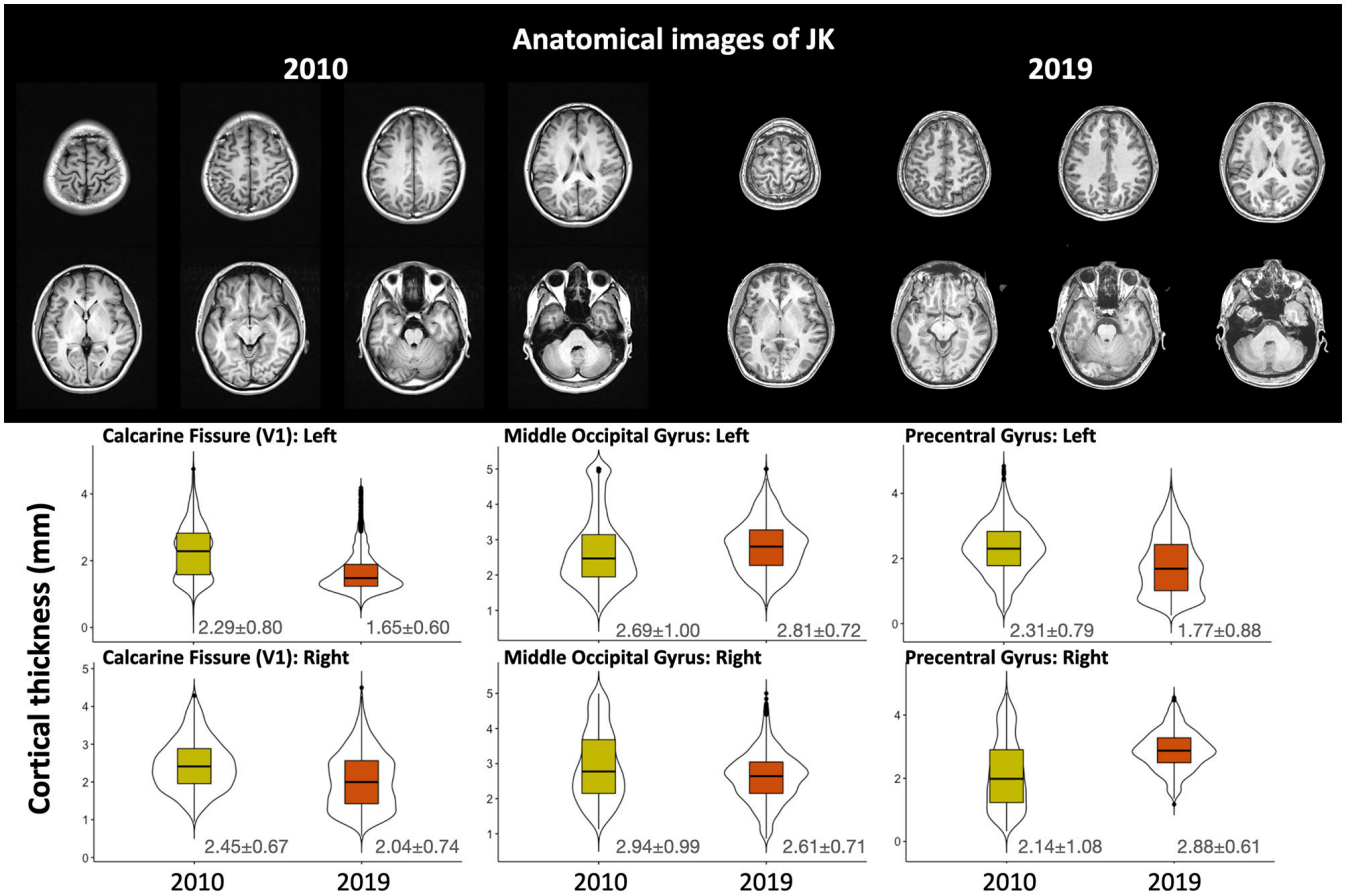
The upper panel shows the T_1_-weighted structural image carried out by the original hospital (2010) and by the Brain and Consciousness Research Center (2019); the lower panel illustrates the cortical thickness through multiple T_1_-weighted images obtained after the accident.

The upper panel of Figure 4 shows the T_1_-weighted structural image from 2010 (left, from another hospital) and 2019 (right, from our Center). Images are shown in their native space, and the lower panel shows the quantified cortical thickness of 3 ROIs (bilateral calcarine fissure, middle occipital gyrus, and precentral gyrus for comparison) through T_1_-weighted images across 2010 and 2019. From the 2019 data, we analyzed cortical thicknesses across eight regions of JK's occipital gyri (bilateral calcarine fissure, inferior occipital gyrus, middle occipital gyrus, and superior occipital gyrus; see Supplementary Figure S2). All were within a reasonable range (2–4 mm), except for the left calcarine fissure (1.65 mm). However, the left precentral gyrus, for comparison, was <2-mm thickness in 2019. Based on these observations, JK's occipital gyri evinced generally preserved cortical thickness, clear evidence that JK's visual cortex did not exhibit MRI-detectable structural impairment after his automobile accident.

**Figure 5:**
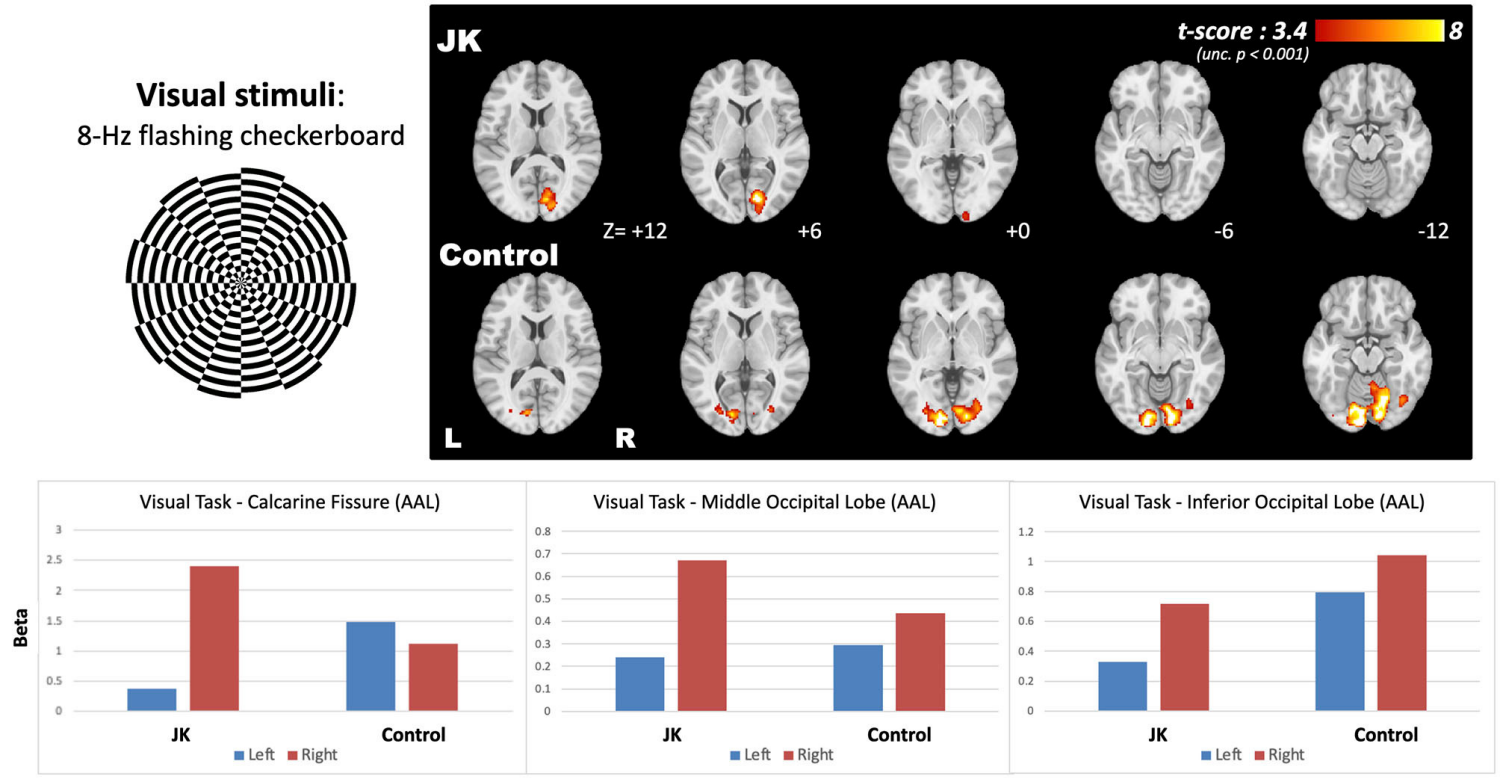
The brain activity following 8-Hz flashing checkerboard visual stimulation, and the bottom panel shows the effect size extracted from the three AAL-based occipital regions.

Figure 5 shows the brain activity maps following 8-Hz flashing checkerboard visual stimulation for both JK and Healthy Control (HC). The normal brain activity (HC) evinced bilateral activation in the visual cortex, but JK exhibited partial brain activity only unilaterally, in the right hemisphere [MNI coordinate: +14, −80, +6]. Left-lateralized brain activity is greatly diminished, implying impairment of visual perception in the right visual field. These quantified results evince the overall reduced brain activity, demonstrating that JK's visual perception is substantially impaired.

**Figure 6:**
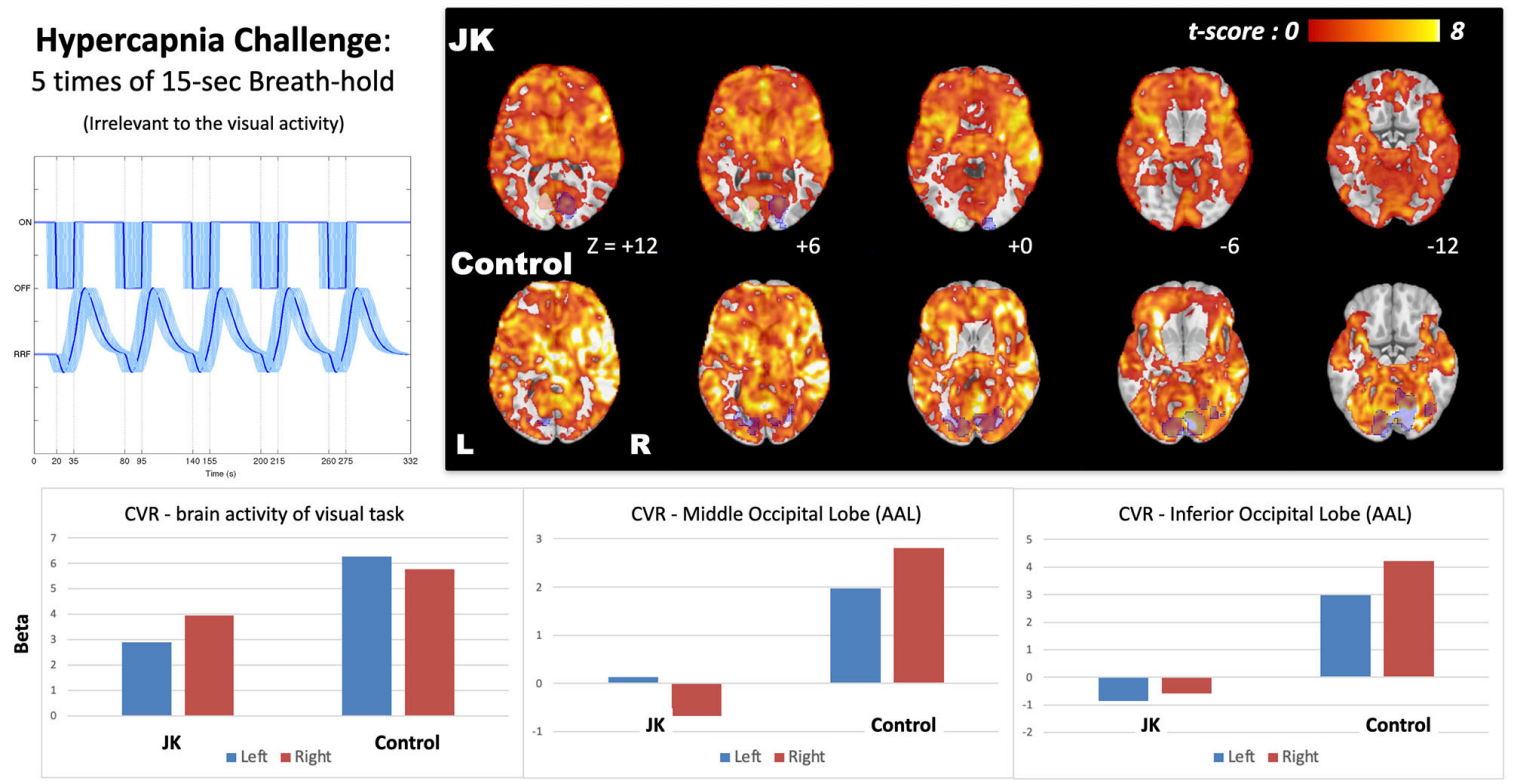
Cerebrovascular reactivity (CVR) map induced by 15-s breath-hold task, a task irrelevant to visual perception; the **bottom** shows the effect size extracted from the brain activity of visual task (blue shaded regions) and two AAL-based occipital regions (middle and inferior). JK's brain activity in the left hemisphere was a mirroring, flipped from the visual activity of the right side (white shaded regions with green stroke).

Figure 6 shows the CVR map generated from the breath-hold task, a task not directly related to visual perception. It reflects transient blood flow surge due to breath-hold-induced hypercapnia in brain tissue. Compared with a general CVR, encompassing all cortical regions, JK evinced observable but weak CVR in the primary visual area in response to the checkerboard task, but showed very weak CVR in other occipital gyri. This impairment is especially prominent in the middle and inferior occipital gyrus (AAL-based ROIs, where the ROI locations were highlighted in Supplementary Figure S2), which implies a neurophysiological deficiency of the transient blood supply in the lateral parts of the visual cortex.

**Figure 7:**
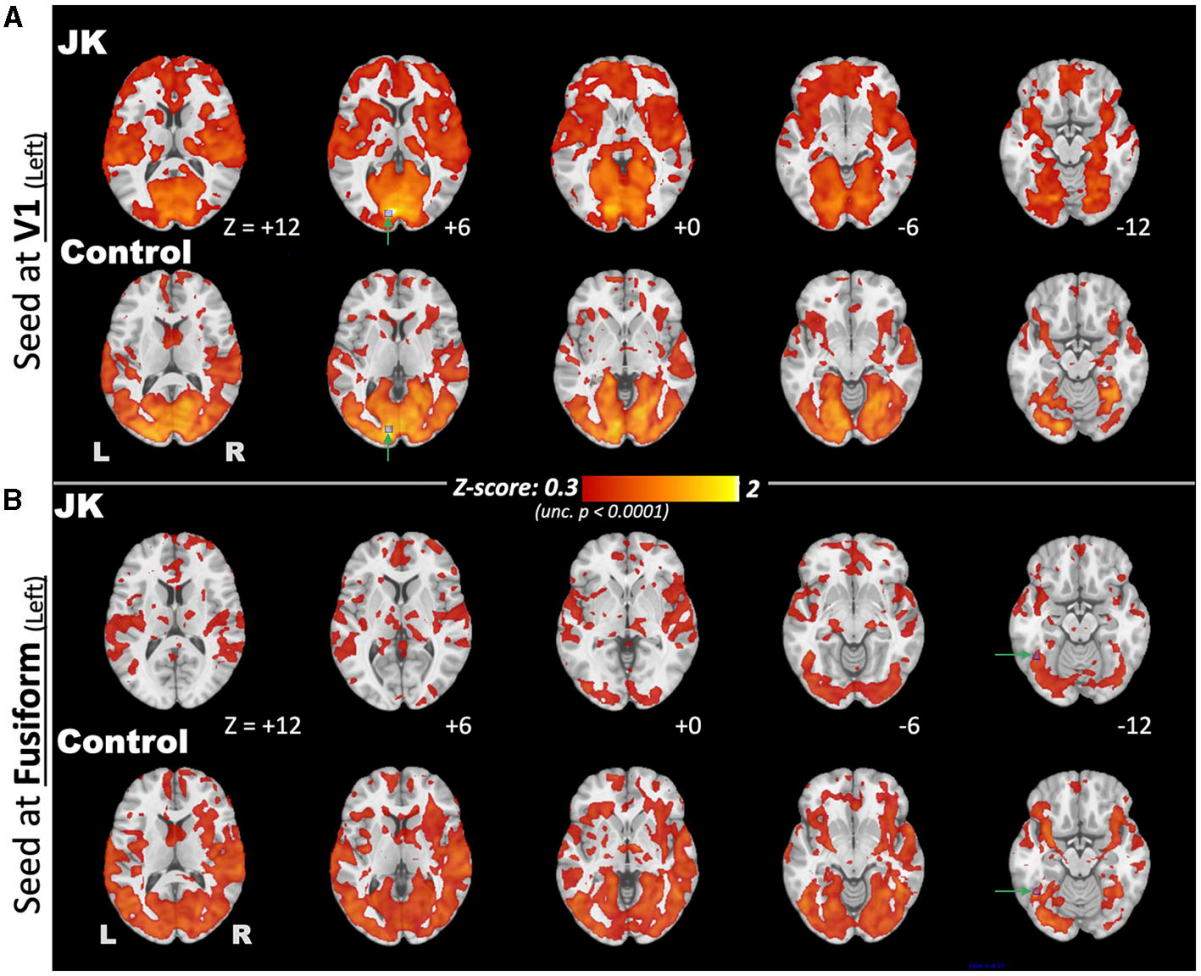
Comparison of eyes-closed (EC) functional connectivity (FC), maps seeded at brain regions **(A)** V1 and **(B)** fusiform, for both JK and Control, where the seed points are highlighted by green arrows and blue squares.

Figure 7 further discloses the comparison of EC FC maps seeded at V1 and fusiform for both JK and HC. Seeded at the left fusiform are (Figure 7, upper), HC's general connectivity to the entire visual network is robust. JK's FC between fusiform and V1, by comparison, is mostly disrupted. This disruption is especially prominent in the middle and superior occipital cortex. When seeded at the left calcarine fissure (V1), the FC extends to the middle occipital gyrus for the HC; this is not the case in JK's FC map (Figure 7, lower). This result indicated that JK's visual network was functionally impaired, lacking sufficient connectivity from V1 and fusiform to other association visual cortices, thereby implying impaired information processing of the object-recognition pathway for visual awareness (66).

## Discussion

JK suffers from bilateral blindsight. Unlike other documented blindsight patients, however, his striate cortex *appears* to be intact, structurally. A former athlete, JK's persisting ability to perform such actions as hitting handballs attracted the attention of people who suspected him of perpetrating fraud. Consequently, in order to adduce evidence of his blindsight and symptoms suggestive of Riddoch Syndrome, while also demonstrating how diagnosis can be rendered in such cases, we carried out an investigation that highlighted use of tasks and functional imaging.

As for JK's conscious residual visual content, it must first be noted that blindsight varies. JK seems to retain some conscious visual experiences: at least he can distinguish light from dark and he sometimes has the experience of bright colors, albeit at a slower speed than is the case for normally sighted people. If his capabilities are mapped onto three-dimensional space—objective capacities, subjective sensation, and “visual” sensation (23)—he has the objective capacity to hit handballs or catch a Frisbee, and he has visual sensations of bright color along with other visually relevant, albeit amodal, sensations. When under bright light with his face pressed up against the page of a book, after a period of intense gazing and angular adjustment, he is able to form light-shadow distinctions. When asked to generalize about his vision, his introspective assessment is either “I don't think that I see anything, but I am not certain” or “I feel something”. Summarizing, his condition approximates Type 2 blindsight (5, 67), although with reference to the three-dimensional model, many of his residual capabilities are more aptly described as amodal “subjective sensations”.

As for the temporal dimension, we employed ERP in an oddball paradigm investigation of P300, comparing JK to a HC. Ordinarily, although the stimuli can be visual or auditory, because we are probing JK's capacity for conscious visual experience, we used visual stimuli, contrasting familiar faces with unfamiliar faces. Indeed, because self-related stimuli are uniquely familiar, playing an important role in the capacity for conscious experience (21, 46), we contrasted JK's self-own-face with unfamiliar faces. For the HC, self and other began to diverge at 247 ms after stimulus onset: that is, upon seeing self-own-face, the P300 component was detected. For JK, on the other hand, we observed no P300, neither for self-own-face nor for unfamiliar faces.

It remains possible that because JK has some—albeit sluggish—conscious percepts, our assumptions about the temporal evolution of EEG signals based on normally functioning brains may not be applicable to him. That is, we must acknowledge the possibility that measurement results for JK's visual perception are not entirely commensurate with those exhibited by healthy controls. Nevertheless, JK's self vs. others difference from 521 ms to 550 ms (Figure 3, bottom) are plausibly interpreted as reflecting his slower, residual, impaired conscious percepts.

If this interpretation is correct, it corresponds to his subjective report for delayed color perception, not unlike what has been reported for scotoma patients' perceptual fill-in process and experience (68). Because JK's small window of differentiation between self and others (29 ms) was only one-tenth the duration exhibited by healthy controls (also with compressed magnitude), however, and because his subjectively-reported delay of perceptual experience is much longer than a few hundred ms, we conjecture that JK's narrow window of difference is less likely to be related to awareness, and more likely attributable to other contaminating sources such as the alpha wave or thermal noise. Currently from Figures 2, 3, we are only able to confirm the initial perception of visual stimuli from N1, which is partly consistent with the task-fMRI activity. Nevertheless, the possibility of a slower, smaller P300 in blindsight patients, one that might be indicative of patients' residual awareness, would be worth investigating, potentially revealing an important but previously overlooked aspect of blindsight.

This particular finding is consistent with the Global Neuronal Workspace Theory (GNWT). The first 200 ms of visual neural activity does not involve formation of conscious percepts. Formation of conscious percepts correlates with events that lag behind stimulus onset by more than 200 ms, as is the case with P300 (52). Just as would be predicted by GNWT, for the HC self and other began to diverge at 247 ms, whereas JK evinced a flattened ERP waveform, with no detectable P300 component for either self-own-face or others' faces.

In this context, it is also worth considering JK's response to static stimuli. Through intense concentration under bright light JK is able to formulate, albeit slowly, conscious percepts of lighted-shadowy distinctions. This too seems consistent with GNWT. GNWT predicts that conscious access proceeds in two successive phases (69): the first, bottom-up phase lasts from ~100 to ~300 ms, as exteroceptive signals climb the cortical hierarchy, non-consciously. Next, modulated by current goals, top-down mechanisms amplify and sustain the bottom-up activity, thereby enabling conscious visual percepts to emerge at ~300–500 ms. Indeed, according to GNWT, the most consistent correlate of conscious visibility just is the broadly distributed, positive component, P300. What appears to be transpiring when JK looks attentively at a brightly lit page is that sufficient bottom-up, non-conscious signals are coupled with top-down attentional mechanisms, so as to precipitate dim, sluggish conscious percepts.

As for the spatial dimension, employing fMRI, perhaps the most striking result obtains for the breath-holding experiment. This task induces a transient, fMRI detectable global hyperemia throughout the cortex; in this way we are able to measure transient hyperemia, the surge in blood flow, in JK's visual cortex. Relative to other cortical regions, JK evinced only weak CVR for most of the occipital gyri, and markedly different than that evinced in the HC (see Supplementary Figure S3, for adjusting different threshold levels in CVR maps). JK's impairment is especially prominent in the middle and inferior occipital cortex, thereby suggesting a neurophysiological deficiency of blood supply for most of the visual cortex. In short, even if current resolution and methodology fail to detect structural impairment, this task cum functional response cohere with JK's subjective report.

Likewise for the spatial dimension, JK and the HC were required to perform another task. With eyes open, they gazed at a full field screen that exhibited two conditions: stimulation and fixation. During stimulation, a black and white circular checkerboard pattern flashed at 8-Hz; during fixation, a static cross pattern was presented. During stimulation, the HC evinced bilateral activation in the visual cortex. For JK, however, brain activity was only detected unilaterally, in the right hemisphere. This finding is consistent with JK's subjective report as well as our behavioral observations: in order for JK to deliberately form a conscious visual percept, under bright light, his left eye approaches to within an inch of a book, a position that he must sustain, along with squinting and angular adjustments, in order that he might gradually form a percept—viz. the contrast between what is bright and what is shadowy.

And, finally, for the spatial dimension, we compared EC FC maps seeded at V1 and at the fusiform for JK and the HC. Seeded at the left fusiform, the HC's connectivity to the entire visual network is robust. JK's FC seeded at fusiform and V1, by comparison, is mostly disrupted, especially in the middle and superior occipital cortex. Seeded at V1, the HC's FC extends to the lateral visual areas (e.g., middle occipital gyrus), unlike JK's. Furthermore, JK's fusiform area (lateral visual area) showed higher connectivity to the prefrontal cortex and salience networks, implying functional reorganization of JK's lateral visual area into functionality unrelated to vision. Even though the lateral visual areas showed irregular connections, however, they may not function well in reacting to transient responses such as the breath-hold challenge. The fMRI evidence converges to suggest that JK's lateral visual areas showed irregular functionality, including: (3) his reaction to the hypercapnia challenge; (4) disrupted connectivity to V1; and (5) additional connectivity to frontal regions. These results indicate that JK's visual network is functionally impaired, lacking sufficient connectivity from V1 to fusiform.

Perhaps due to JK's impaired visual function, non-vision functional connectivity provides a possible compensatory network reorganization. Indeed, Supplementary Figure S4 suggest just such a reorganization of JK's brain-networks subsequent to the accident. In particular, take note of the default-mode and the sensorimotor networks.

Based on JK's apparent N1 component (Figure 2) and limited V1 activation (Figure 5), he might yet preserve partial visual reception from V1; however, V1-based visual processing is insufficient to support the entirety of visual perception. Based on JK's (a) weak P300 in face recognition (Figures 2, 3), (b) minimal CVR in lateral visual area (Figure 6), and (c) low fusiform connectivity (Figure 7B), we speculate that JK's functional impairments in the lateral visual areas (near the fusiform), block the feedforward pathways of the visual network, impeding his ability to integrate visual inputs into an intact visual perception. This in turn implies impaired information processing in the object-recognition pathway. This finding too is consistent with JK's subjective report: when able to form a conscious visual percept of a handball bouncing toward him, he reports a patch of color that lacks a distinctive shape.

Can anymore be said about the conscious vision-related sensations that JK does experience? Bright light lingers, after he has entered a tunnel, and the dark persists after emergence. The yellow quale of a handball lingers before his eyes, after the ball has already hit him in the face. Although this awaits further investigation, JK might be suffering from a form of akinetopsia, visual motion blindness (70, 71), at least in the sense that for JK color and motion seem to dissociate (72), resulting in the appearance of a color patch that hovers motionless, as though viewing the individual frame of a film. An alternative possibility is that conscious percepts are formed before they are experienced as belonging to self, as has been associated with hypometabolism in the parieto-occipital regions (73, 74).

Does the finding that JK's conscious percepts seem to operate on an intrinsic delay setting suggest anything about the nature of conscious sensory experience? Although there is no grand consensus concerning the nature of consciousness on the horizon (75), many investigators agree on the following: (1) the neural substrate of conscious sensory activity is more sluggish than is the neural substrate of nonconscious sensory activity (52). (2) Conscious experience is a *duration*, an extended “specious present,” that is experienced as this-present-moment (76, 77). And, (3) conscious experience does not represent the world as it is; instead, it is an imagined model, possibly Bayesian, that enables generally successful navigation of our environment (78). This model has sometimes been called a “controlled hallucination”—to distinguish it from dreams, psychotic, or psychedelic experiences—and to underscore that although it is an imaginary representation, when we are in a normal waking state, the world we hallucinate is constrained by the external world. JK's visual experiences are a reminder that for our imaginary representations of the world to be properly constrained, mechanisms regulating our perception of time can neither be too sluggish, nor allow for too much variation in the temporal span of the present moment. We speculate that JK's conscious experience is inadequately “controlled” either because his visual experiences are excessively sluggish or his sensory window has been foreshortened to such a degree that fewer changes are admitted to his specious present. This suggests that if consciousness has a function, one function of our “controlled hallucination” is to coordinate time perception with objective time. In a word, coordinate entropy in the brain with entropy in the external world (79).

A potential limitation concerns our efforts to confirm the absence of detectable structural damage to the occipital gyri. Toward this end we quantified cortical thickness from four different scans, taken at four different sites, only one of which was conducted by our Brain and Consciousness Research Center. Consistent with a premise of our investigation—a functional methodology is best suited to detecting evidence of JK's blindsight—we found that JK's occipital gyri exhibited either preserved or increased cortical thickness. There was no evidence of reduction. But the T_1_-weighted anatomical images that we used were collected from different MRI sites, so we must acknowledge the possibility that cross-site variability could yield inconsistent results for cortical thickness.

Nevertheless, the longitudinal tracing evidence that we adduced strongly suggests that JK's visual cortex exhibited little or no reduction. Second, the eye-tracker enabled us to confirm participant compliance with the visual task. But the eye-tracker record was not stored, thereby precluding analysis of gaze direction during the stimuli onset. Because JK's performance on the visual tasks did not convince the judiciary of JK's cognitive visual impairment, however, we designed the full-field flashing checkerboard task to determine JK's primary visual cortex function. Our observation of JK's V1 activity under the checkerboard stimuli resembled the prior investigation, which provided the cross-cite consistency under the same task. Following this confirmation of vision-relevant evidence, we proceeded to put more emphasis on vision-irrelevant task performances, such as breath hold and the resting state.

The constrained conditions and low success rate for Frisbee catching and hitting a pitched baseball that JK reported in our most recent interview, could cause one to wonder about the degree to which these performances might depend on auditory percepts, even perhaps echolocation (80). Nevertheless, given that the speed of light is 186,000 miles per second while the speed of sound in air is 0.2 miles per second, and given that hitting a baseball requires extremely quick perception-action coupling (81, 82), it is probable that JK's successful performance is mostly due to the processing of visual signals. The requisite agility gives credence to JK's subjective report that sound is primarily used just for directional orientation and gauging release time of the projectile.

Another limitation of our study is that we were unable to carry out some among the behavioral probes—visual discrimination assessments that can provide important data on wavelength, motion, and orientation—probes used in previous investigations of blindsight. Unfortunately, JK's case was brought to our attention more than a decade after his TBI occurred and all previous investigations of his condition were clinical, not methods designed for basic science investigations. Accordingly, our focus was to build on those clinical assessments and design novel, *functional* probes in temporal and spatial dimensions, in order to help explain the mismatch between JK's subjective report and *structural* scans of his striate cortex that employed standard clinical parameters. The forensic matters pertaining to JK's case also contributed to our decision to concentrate on this novel approach to blindsight. Nevertheless, had we been able to use standard visual discrimination tasks when assessing the trajectory of JK's blindsight, we believe it would have been of great value, especially since unlike other blindsight patients (83), JK's symptoms become aggravated not ameliorated. A longitudinal study of his performance on visual discrimination tasks might contribute to explaining the unusual course of his visual impairment.

Although we do not claim that the case of JK constitutes a counter-example to the biological principle that structure determines function, JK seems to be unique among blindsight patients, in that multiple scans evince no evidence of striate structural damage, possibly due to the limited resolving power of three Tesla scanners. Given the absence of structural evidence, we are presented with a legal, clinical, and scientific conundrum—how to determine whether he does indeed suffer from blindsight. For that purpose we designed a series of investigations for both temporal and spatial dimensions in order to confirm that objective measures comport with JK's subjective report. As a matter of fact then, task-based functional evidence from temporal-spatial domains does help account for the distinctive character of JK's blindsight, including its various nuances.

To conclude, our study demonstrates how multi-modal, task-based, neuroimaging methodologies can converge to adduce functional evidence that coheres with a blindsight patient's description of conscious experience. This approach can provide clinicians and forensic neuroscientists with a novel set of tools to aid in the diagnosis of blindsight, Riddoch's Syndrome, and possibly other types of cortical blindness or severe visual impairment. What is more, JK's condition is distinctive among reported cases of blindsight in that residual awareness impedes motor or athletic performance. This rarity, perhaps even uniqueness, stands to contribute substantially to efforts aimed at achieving an empirically-based, comprehensive understanding of blindsight. And teasing apart the capabilities of nonconscious from conscious sensory states is one means whereby possible functions of consciousness can be identified.

## Data availability statement

The raw data supporting the conclusions of this article will be made available by the authors, without undue reservation.

## Ethics statement

The studies involving humans were approved by Taipei Medical University-Joint Institutional Review Board (N202111025). The studies were conducted in accordance with the local legislation and institutional requirements. The participants provided their written informed consent to participate in this study. Written informed consent was obtained from the individual(s) for the publication of any potentially identifiable images or data included in this article.

## Author contributions

TL, PT, and CW wrote the manuscript. CW, PT, T-HL, and TL designed the study. PT, CW, and TL collected the data. CW, PT, Y-CK, T-HL, and TL analyzed the data. All authors consulted on revising and approving the manuscript.
