# Supplementary material for: Functional blindsight and its diagnosis
Source: Front Neurol. 2024 Feb 7;15:1207115. doi: 10.3389/fneur.2024.1207115 (PMC10879618; doi:10.3389/fneur.2024.1207115)
Supplement: Supplementary file 1 [file Data_Sheet_1.PDF]

Supplementary Materials

Functional Blindsight and Its Diagnosis

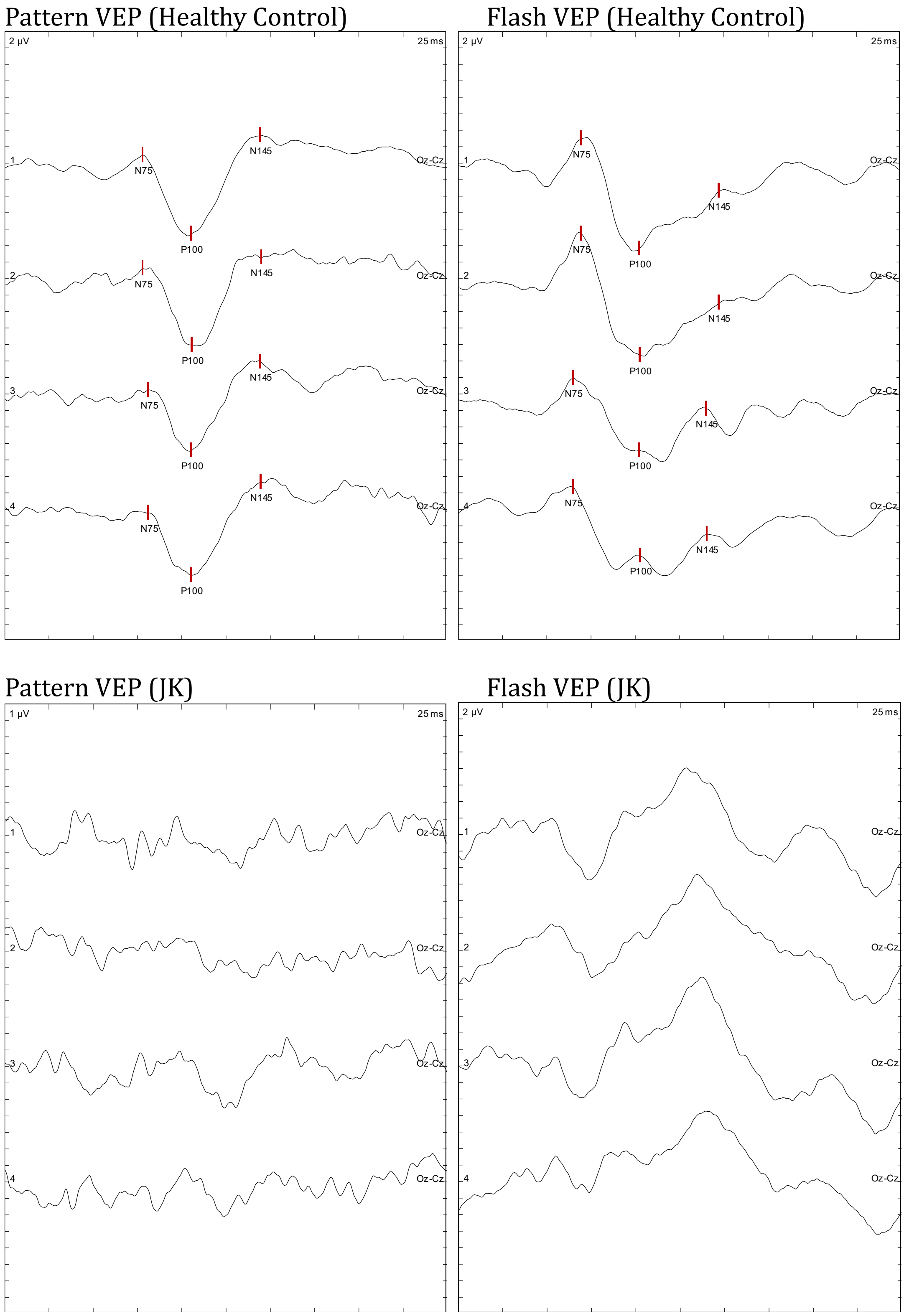

Figure S1 Pattern and flash visual-evoked potentials (VEP) from HC and JK. Unlike HC, the N75, P100, and N145 of JK could not be identified (spatial frequency of Pattern VEP: 0.435 cycles per degree).

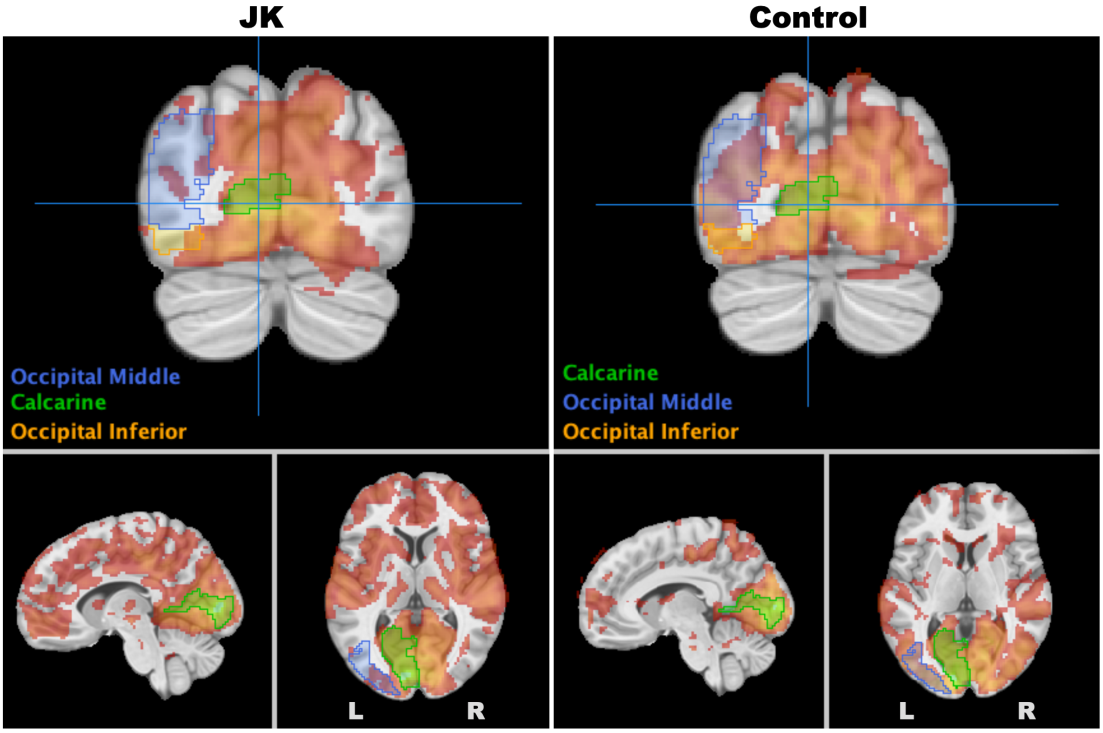

Figure S2 Demonstration of the region-of-interest (ROI) selections from the AAL template. The left panel is the three views of ROIs overlaid on JK's visual-network connectivity map (Z > 0.3, seeding at the left primary visual cortex [-10, -88, 5]) and standard anatomical image, and the right panel is the same ROIs overlaid on the control's visual-network connectivity map. In the quantitative evaluations of the visual areas, we prescribed the middle occipital lobe (blue regions, AAL#51), inferior occipital lobe (orange regions, AAL#53), and the calcarine fissure as the primary visual area (V1, green regions, AAL#43). In this map, we only demonstrated the ROIs in the left hemisphere; in Fig.5 and Fig.6, we also used the contralateral side of each ROI in the right hemisphere (AAL#52, #54, and #44) for comparisons.

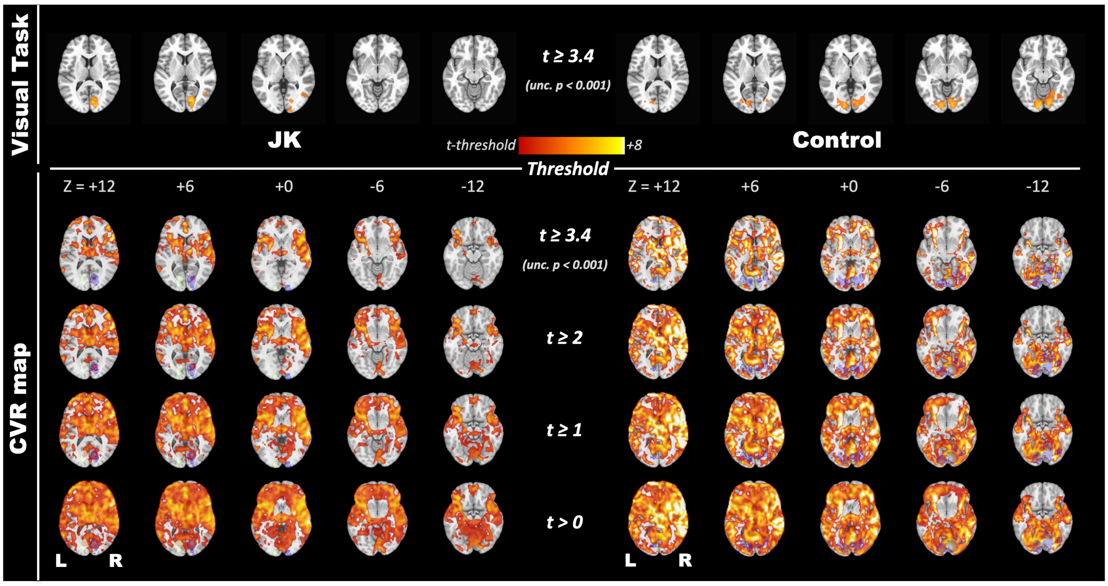

Figure S3 The breath-hold cerebrovascular reactivity (CVR) maps at different threshold levels of t scores. The brain activity of checkerboard visual stimuli was listed in the top row for comparison. With the elevated t thresholds, it is prominent that JK has relatively weak CVR in the posterior brain under the challenge of breath-hold task, as compared with the control.

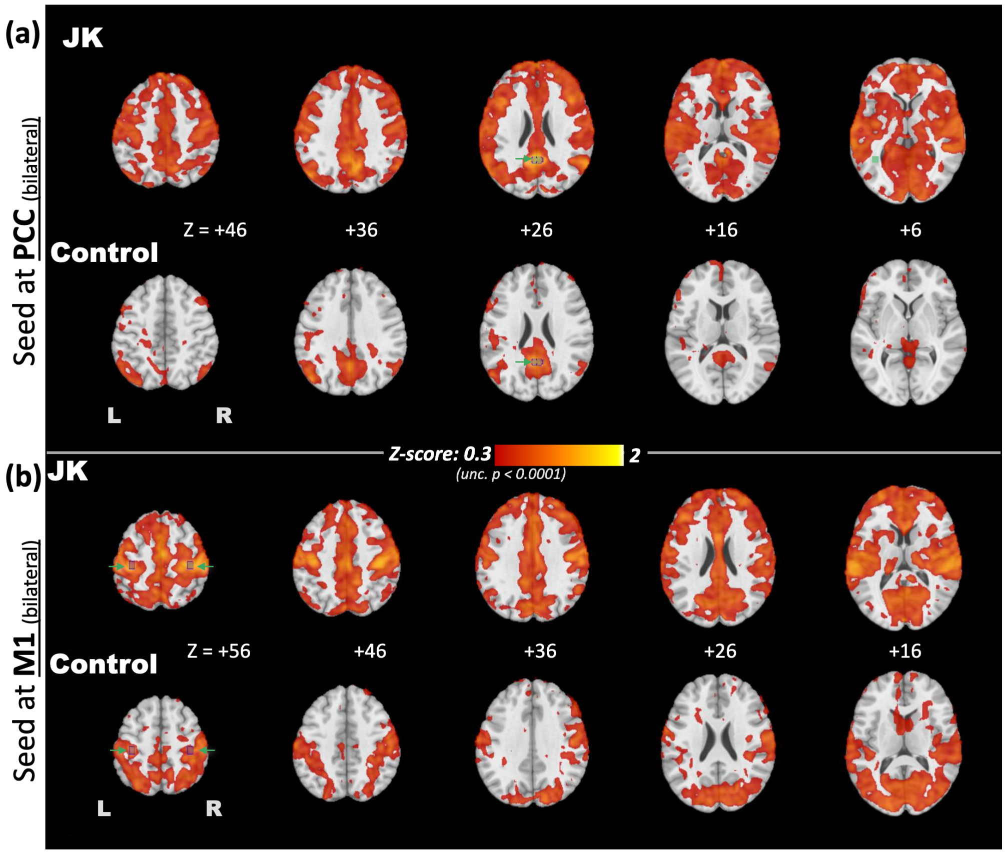

Figure S4 The resting-state functional connectivity of default-mode network (a, DMN, seeding at the bilateral posterior cingulate cortex [±3, -53, 26]) and sensorimotor network (b, SMN, seeding at the bilateral primary motor cortex [±36, -25, 57]) for both JK and the control. The seed areas were denoted with blue boxes with green arrows. As compared to those of the control, the DMN and SMN connectivity patterns of JK showed widespread connections to the frontal regions and less connections to the high-order visual areas beyond the primary visual cortex.

Reference of seed location:

V1: [-10, -88, 5]: Wu et al. Neuroimage 2009;45:694-701.

Fusiform: [-44, -52, -14]: Ward et al. Hum Brain Mapp. 2014;35(3):1061–1073.

PCC: [±3, -53, 26]: Van Dijk et al. J Neurophysiol 2010;103:297-321.

Motor [±36, -25, 57]: Van Dijk et al. J Neurophysiol 2010;103:297-321.
